# Patients’ Experiences of Nurse-Led eHealth Interventions for Chronic Heart Failure: Qualitative Systematic Review and Meta-Synthesis

**DOI:** 10.2196/82714

**Published:** 2026-07-06

**Authors:** Yifan Zhang, Yanjie Ma, Wenrui Han, Yilong Song, Zhixian Wang, Xingda Jin, Wenlin Cheng, Shukun Yu, Rui Li

**Affiliations:** 1 School of Nursing, Beihua University, Jilin, China; 2 Wuxi School of Medicine, Jiangnan University, Wuxi, China; 3 Graduate School, Shanghai University of Traditional Chinese Medicine, Shanghai, China; 4 Department of Nursing, Tongren Hospital Shanghai Jiao Tong University School of Medicine, Shanghai, China; 5 Department of Nursing, The Affiliated Hospital of Beihua University, No 12 Jiefang Middle Road, Chuanying District, Jilin, 132013, China, 86 432 62166000; 6 Department of Nursing, Tongji University Affiliated Shanghai Pulmonary Hospital, Shanghai, China

**Keywords:** chronic heart failure, nurse-led, eHealth, patient experience, qualitative research, systematic review, meta-synthesis

## Abstract

**Background:**

Chronic heart failure (CHF) is a major chronic condition in the context of global population aging and is associated with high prevalence, frequent rehospitalization, and high mortality. Self-management is widely recognized as an important factor influencing prognosis and quality of life among patients with CHF. Nurse-led eHealth interventions have been increasingly used in postdischarge CHF management and have shown potential for improving adherence and health-related behaviors. However, existing studies have predominantly focused on objective outcomes, and limited systematic synthesis has examined patients’ subjective experiences, which may restrict the patient-centered optimization and sustainable implementation of these interventions.

**Objective:**

This study aimed to systematically synthesize patients’ experiences of nurse-led eHealth interventions for CHF, identify facilitators and barriers to engagement and implementation, and inform the patient-centered optimization of intervention design through a qualitative systematic review and meta-synthesis.

**Methods:**

This qualitative systematic review and meta-synthesis was conducted in accordance with the Joanna Briggs Institute methodology for qualitative systematic reviews. A comprehensive search was performed in PubMed, Web of Science, Embase, the Cochrane Library, CINAHL, CNKI, WanFang, and VIP from database inception to April 30, 2026, supplemented by manual searches of the reference lists of the included studies. Following methodological quality appraisal, the findings of the included studies were extracted and synthesized using thematic synthesis.

**Results:**

A total of 23 studies involving 424 patients with CHF were included, comprising 17 qualitative studies and 6 mixed methods studies. Four synthesized themes and 12 subthemes were generated: (1) patient empowerment and enhanced self-management, (2) sense of security and continuity of care under professional support, (3) variations in acceptance and emotional responses, and (4) barriers and challenges in implementing eHealth interventions.

**Conclusions:**

Nurse-led eHealth interventions may provide important support for CHF management from the patient perspective by promoting empowerment and extending care support beyond hospital settings. The findings suggest that future intervention design should better address patient heterogeneity, technological usability, and long-term sustainability. Further efforts are needed to strengthen individualized adaptation and technological optimization to enhance the accessibility, acceptability, and patient-centered implementation of these interventions.

## Introduction

With accelerating population aging and the growing burden of chronic diseases, chronic heart failure (CHF) has become a major public health concern worldwide [1]. CHF is a complex clinical syndrome resulting from structural or functional cardiac abnormalities that impair ventricular filling and/or ejection, and its prevalence increases substantially with advancing age [2]. Data from the Global Burden of Disease Study 2021 indicate that more than 64 million people worldwide are living with CHF, and both prevalence and mortality continue to rise [3]. Beyond its high prevalence and substantial mortality risk, CHF is also characterized by progressive disease deterioration. After diagnosis, patients may experience clinical decline over several years, and the 5-year survival rate remains low, comparable to that of certain malignancies [4]. Frequent episodes of acute decompensation can lead to repeated hospitalizations, substantially compromising patients’ quality of life and functional status and imposing a considerable burden on health care systems [5-7]. Therefore, delaying disease progression, reducing acute exacerbations, and maintaining long-term continuous health management after discharge are critical priorities in CHF care.

In CHF management, self-management is widely recognized as an important determinant of prognosis [8]. Effective self-management encompasses medication adherence, dietary and sodium restriction, appropriate physical activity, daily monitoring of body weight and symptom changes, and timely recognition of and response to warning signs [9,10]. In practice, however, insufficient disease-related knowledge, low self-efficacy, and a lack of continuous professional guidance often make it difficult for patients to sustain these self-care behaviors, which may adversely affect clinical outcomes [1,8-10].

To enhance patients’ self-management capacity, nurse-led interventions have become an important component of chronic disease management [11-14]. Nurses have professional expertise and accessibility in health education, medication guidance, symptom monitoring, and follow-up support, enabling them to improve patients’ self-management capacity through individualized guidance and psychological support [9,13]. With the continued development of information technology, eHealth interventions provide new opportunities for extending nurse-led chronic disease care [14]. eHealth generally refers to health services and management activities delivered through information and communication technologies, encompassing mobile health, telehealth, telemonitoring, web-based platform support, telephone or video follow-up, and digital health education [15]. Given the overlap among these related terms in the existing literature, this review uses eHealth as an overarching concept. Compared with conventional face-to-face nursing care, nurse-led eHealth interventions can reduce temporal and geographical constraints, allowing patients to continue receiving professional support after discharge [14,16]. Such interventions may help improve self-management adherence and support the implementation of health behaviors and may contribute to reduced rehospitalization risk and improved quality of life [17].

The application of eHealth technologies in CHF management has become an increasing focus of research [18]. Existing evidence suggests that nurse-led interventions delivered through digital platforms can improve patients’ treatment adherence and disease-related knowledge, support health-promoting behaviors, and enable early identification of changes in clinical condition, which may contribute to improved patient outcomes and reduced rehospitalization risk [16,19-21]. In this process, nurses typically serve as core implementers and coordinators by integrating multidisciplinary resources and using digital platforms to provide individualized nursing guidance and health management services, forming a technology-integrated care model characterized by “nurse leadership plus technological support” [20,21]. However, existing studies have primarily focused on objective outcomes, with relatively limited attention to patients’ subjective experiences. In particular, patients’ lived experiences and user feedback during the intervention process remain insufficiently synthesized, including key dimensions such as acceptance of remote monitoring tools, perceived convenience of and barriers to technology use, and feelings of security or anxiety associated with the intervention [22,23]. Even when an intervention demonstrates favorable effects on clinical indicators, poor user experience or difficulty sustaining patient engagement may limit its sustained use and feasibility for wider implementation [24].

Qualitative research provides an important approach for understanding patients’ subjective experiences of eHealth interventions. Several studies have examined the emotional and behavioral responses of patients with CHF during nurse-led remote care and have revealed diverse experiences [25-27]. For example, patients undergoing remote monitoring often report an enhanced sense of security and psychological reassurance, whereas some encounter technical difficulties during initial use and experience anxiety or uncertainty [25]. These findings suggest that nurse-led eHealth interventions may empower patients and strengthen self-efficacy, whereas technological barriers or insufficient support may create difficulties in adaptation [27]. Although these qualitative studies provide important insights, they are often limited by small sample sizes and varied study contexts, leaving the relevant findings relatively fragmented. This fragmentation limits an integrated understanding of patients’ intervention experiences, facilitators, and barriers, and may constrain the development of practice-oriented strategies for intervention optimization.

Therefore, this study used a qualitative systematic review and meta-synthesis to integrate patients’ experiences of nurse-led eHealth interventions for CHF. By including qualitative studies and mixed methods studies with qualitative findings, this review focused on the patient perspective and synthesized patients’ perceptions, attitudes, and feedback throughout the intervention process. It further identified key facilitators and barriers to intervention implementation to inform the development of more adaptive and individualized intervention strategies and support the optimization of patient-centered remote nursing services.

## Methods

### Study Design

This study used a qualitative meta-synthesis approach [28] to synthesize the experiences, perceptions, and perspectives of patients with CHF receiving nurse-led eHealth interventions. The study was conducted in accordance with the methodological procedures for qualitative evidence synthesis developed by the Joanna Briggs Institute (JBI) to enhance the methodological rigor, transparency, and traceability of the evidence generation process [29]. This review was reported in accordance with the PRISMA (Preferred Reporting Items for Systematic Reviews and Meta-Analyses) checklist, which is provided in Checklist 1.

### Search Strategy

A comprehensive search strategy was developed based on 4 core concepts: nurse-led care, CHF, eHealth interventions, and qualitative research or patient experiences. The search terms included, but were not limited to, the following: “nurse-led,” “nurse managed,” “nurse-directed,” “nurse-delivered,” and “nurs*” for nurse-led care; “heart failure,” “cardiac failure,” “myocardial failure,” “congestive heart failure,” “left-sided heart failure,” “right-sided heart failure,” and “heart decompensation” for CHF; “telemedicine,” “telehealth,” “telecare,” “telemonitor*,” “telecoaching,” “telenursing,” “eHealth,” “e-health,” “mHealth,” “m-health,” “gerontechnology,” “telerehabilitation,” “e-rehabilitation,” “telecommunication,” “videoconferenc*,” “teleconferenc*,” “internet,” “computer*,” “mobile,” “phone*,” “smartphone*,” “telephone*,” “tablet*,” “email,” “e-mail,” “SMS,” “app*,” “social media,” “wireless,” “virtual,” “remote,” “distant,” and “technolog*” for eHealth interventions; and “qualitative,” “focus group*,” “interview*,” “experienc*,” “attitud*,” “feel*,” “respons*,” “perspectiv*,” “opin*,” “phenomenolog*,” “lived experience*,” “narrative*,” “ethnograph*,” “grounded theory,” and “content analysis” for qualitative research and patient experiences. Search terms within each concept were combined using the Boolean operator “OR,” and the 4 concept blocks were combined using “AND.” No “NOT” operator was applied. Both controlled vocabulary terms and free-text keywords were used, and the search strategy was adapted to the indexing system of each database. Quotation marks were used consistently for all search terms, and truncation symbols (*) were applied to capture lexical variations. No start-date restriction was applied, and all databases were searched from inception to April 30, 2026, to ensure comprehensive retrieval of the available evidence. The full reproducible search strategies for each database are provided in Multimedia Appendix 1 in accordance with PRISMA-S (Preferred Reporting Items for Systematic Reviews and Meta-Analyses Literature Search Extension) recommendations. Two reviewers (YZ and YM) independently conducted the database searches and cross-checked the search results to ensure accuracy and consistency.

### Inclusion and Exclusion Criteria

Based on the population, phenomenon of interest, context, and study design framework, this review established explicit inclusion and exclusion criteria, as detailed in Table 1.

**Table 1.** Inclusion and exclusion criteria.

	Inclusion criteria	Exclusion criteria
P (population)	Adults aged ≥18 years with a confirmed diagnosis of chronic heart failure (CHF). No restrictions on gender, ethnicity, education level, or social background.	Patients without CHF. Participants younger than 18 years of age. Studies focusing on health care professionals or caregivers. Animal studies.
I (phenomena of interest)	Nurse-led eHealth interventions, including but not limited to remote monitoring, mobile apps, online education, telephone or video follow-ups, etc. Focused on patients’ subjective experiences, perceptions, attitudes, needs, facilitators, or barriers.	Interventions not led by nurses (eg, physician-led or technician-led). Interventions outside the scope of eHealth. Studies not centered on patients’ experiences (eg, focused on nurses or caregivers).
Co (context)	Real-world settings involving nursing care or self-management, including home, outpatient, community, and hospital environments. Interventions conducted in actual care scenarios.	Simulated or virtual settings. Nonclinical interventions. Studies only evaluating effectiveness without addressing experiential data.
S (study design)	Qualitative studies or mixed methods studies with qualitative results. Methodologies were not limited to ethnography, phenomenology, qualitative inquiry, action research, discourse analysis, and grounded theory.	Papers without full text. Quality grade C.
Language	Studies published in English or Chinese.	Studies published in languages other than English or Chinese.

### Study Selection

All retrieved records were imported into EndNote (version 21; Clarivate) for reference management and deduplication. Following deduplication, 2 reviewers independently screened the titles and abstracts against the predefined eligibility criteria to identify potentially relevant studies. After excluding records that did not meet the eligibility criteria, the full texts of potentially eligible papers were independently assessed to determine final inclusion. Any disagreements arising during the selection process were resolved through discussion and consensus; when necessary, a third reviewer was consulted for arbitration.

### Quality Appraisal

The methodological quality of all included studies was assessed using the JBI Critical Appraisal Checklist for Qualitative Research [30]. This tool comprises 10 items evaluating key methodological domains, including philosophical underpinnings, researcher positioning and influence, participant recruitment, transparency in data collection and analysis, and ethical considerations. Each item was rated as “yes,” “no,” “unclear,” or “not applicable.”

The appraisal was independently conducted by 2 researchers (YZ and YM) with experience in systematic reviews. Any discrepancies in scoring were resolved through discussion and consensus, with arbitration by a third researcher when necessary. Based on the extent to which the appraisal items were met, the research team made an overall judgment of methodological quality. Studies that generally met the appraisal criteria and had minimal methodological limitations were rated as grade A. Studies with some reporting deficiencies that did not compromise overall credibility were rated as grade B. Studies in which most key items failed to meet the criteria, or that had evident methodological limitations or substantial concerns regarding methodological quality, were rated as grade C and excluded. Ultimately, only studies rated as grade A or grade B were included to enhance the credibility and interpretive value of the synthesized evidence.

### Data Extraction

Data extraction was independently performed by 2 researchers after full-text review to promote a systematic and consistent extraction process. Using a prespecified data extraction form, the research team extracted information from the included studies, including author, year of publication, country or region, study setting, sample size and participant characteristics, study design, study aims, data collection methods, duration of interviews or data collection, data analysis methods, and the main findings reported in each study. For studies that included patients, caregivers, or health care professionals, only experiential data clearly attributable to patients with CHF were extracted. For mixed methods studies, only qualitative findings that could be clearly separated from the quantitative results were extracted. After the initial comparison of extracted data, discrepancies were reviewed by a third researcher to enhance data accuracy and completeness. The final extracted data were used for subsequent meta-synthesis and interpreted in conjunction with the methodological quality appraisal results.

To avoid duplicate inclusion of the same participant sample, the research team compared author groups, study settings, sample sources, recruitment periods, intervention program names, and study characteristics during full-text screening and data extraction to identify potential duplicate publications from the same research project or participant sample. If multiple papers were based on the same sample, only the report providing the most complete information and most closely aligned with the aim of this review was retained. After verification, no sample overlap was identified among the studies ultimately included, and the participant samples reported in each study were considered independent.

### Meta-Synthesis

This study combined meta-aggregation and thematic analysis to synthesize the experiences and perspectives of patients with CHF receiving nurse-led eHealth interventions, as reported in qualitative studies and mixed methods studies with qualitative findings. Two researchers independently and repeatedly read all included studies and identified findings explicitly presented by the authors of the primary studies. The credibility of these findings was assessed in relation to the accompanying original participant quotations. On this basis, findings with similar meanings were grouped into categories and further integrated through team discussions into overarching themes to capture the core content and internal logic of patients’ experiences. Throughout the synthesis process, emphasis was placed on the relationships among findings and the clarity of the conceptual structure to support an interpretation that reflected patients’ shared experiences and practical challenges. The research team maintained analytic records during the synthesis process and used repeated comparison and team discussions to enhance the transparency, traceability, and interpretive rigor of the analytic pathway.

### Confidence Assessment of Meta-Synthesis Findings

This study assessed the level of confidence in the evidence for the final synthesized themes using the Confidence in the Evidence from Reviews of Qualitative Research (ConQual) approach developed by the JBI [31]. This approach begins with an initial rating of “high,” followed by downgrading based on 2 core domains: the methodological dependability of the primary studies and the credibility of the study findings. The final level of confidence is then determined and categorized as high, moderate, low, or very low.

Methodological dependability was assessed using 5 critical items from the JBI Critical Appraisal Checklist for Qualitative Research, reflecting the overall rigor and methodological conduct of each included study. If 4 or 5 of these items were rated “yes,” no downgrading was applied; if 2 or 3 were rated “yes,” the confidence rating was downgraded by 1 level; and if only 1 or none was rated “yes,” the confidence rating was downgraded by 2 levels.

The credibility of findings focused on the alignment between the researchers’ interpretations and the original participant quotations across the synthesized themes. Findings were categorized as “unequivocal,” “credible,” or “unsupported.” No downgrading was applied for themes supported solely by unequivocal findings; themes supported only by credible findings were downgraded by 2 levels; and themes with unsupported findings were downgraded to very low.

Each synthesized theme was evaluated using the ConQual approach to present the strength and trustworthiness of the qualitative evidence and to support interpretation of the robustness and applicability of the review findings.

### Ethical Considerations

As this study is a systematic review and meta-synthesis that does not involve the collection of primary data, ethics approval was not required. All included studies had received prior ethical clearance and obtained informed consent from participants.

## Results

### Study Selection

**Figure 1.**
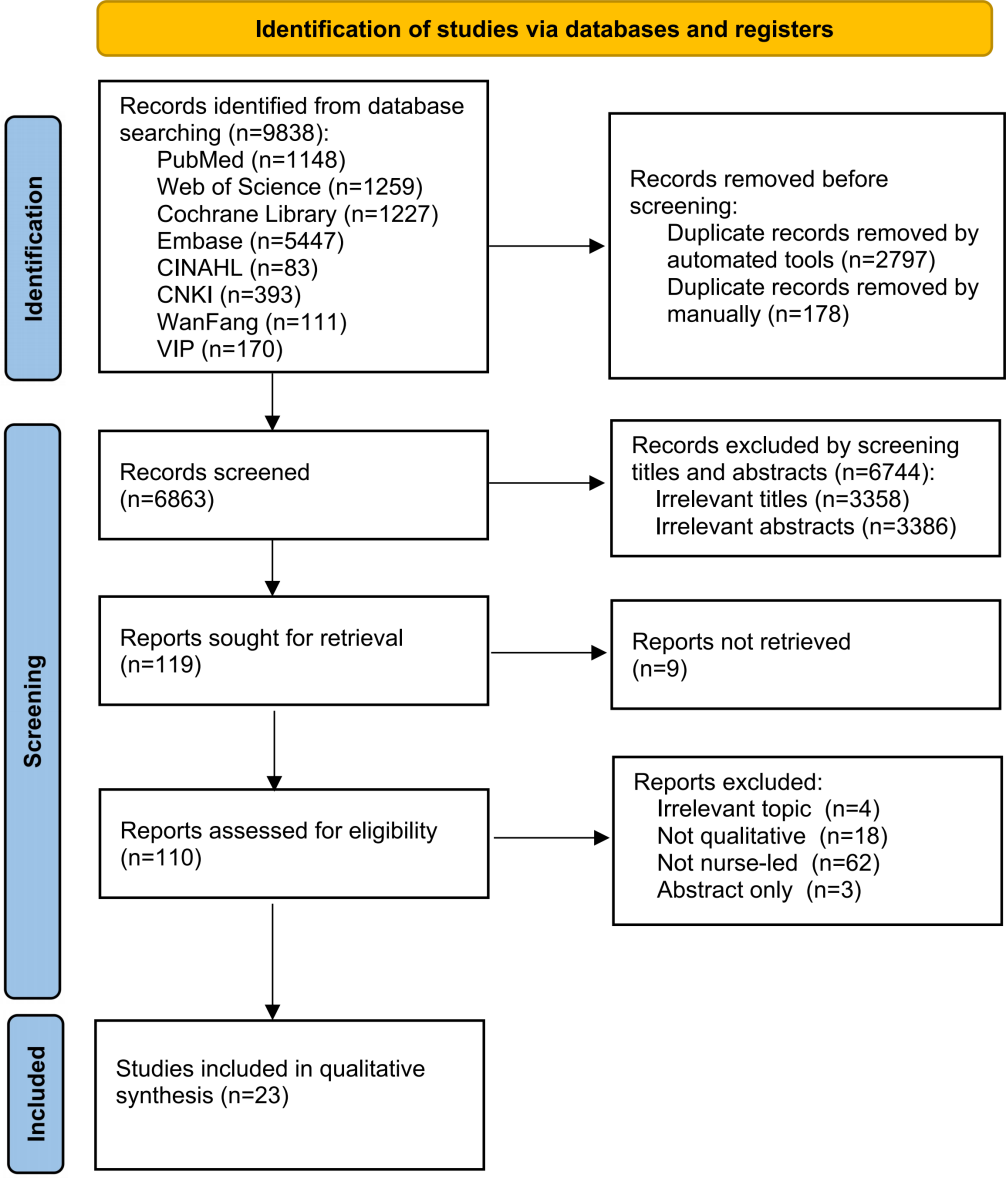
Search flow diagram.

The database search yielded 9838 records. After deduplication and screening of titles and abstracts, 110 papers underwent full-text review. Ultimately, 23 studies [25-27,32-51] met the inclusion criteria and were included in the synthesis (Figure 1). The included studies were conducted in the United States (n=5), Sweden (n=4), China (n=3), Canada (n=3), Norway (n=2), the United Kingdom (n=1), Singapore (n=1), South Korea (n=1), Japan (n=1), Italy (n=1), and Lithuania (n=1). These studies involved a total of 424 patients with CHF. The study designs comprised 17 qualitative studies and 6 mixed methods studies. Detailed characteristics of the included studies are presented in Multimedia Appendix 2 [25-27,32-51].

### Quality Appraisal Results

According to the JBI Critical Appraisal Checklist for Qualitative Research, the included studies generally demonstrated good methodological quality. Two studies were rated as grade A [42,45], and 21 studies were rated as grade B [25-27,32-41,43,44,46-51]. However, reporting deficiencies remained in several appraisal items. Specifically, 7 studies [26,32,40,41,44,46,48] did not clearly report the congruity between the stated philosophical perspective and the research methodology; 3 studies [32,40,46] did not clearly describe the researchers’ cultural or theoretical positioning; and 20 studies [25-27,32-39,41,43,44,46-51] did not describe the influence of the researchers on the research or the influence of the research on the researchers. The remaining appraisal items were generally adequately reported. Overall, the included studies demonstrated good congruity in terms of research questions or objectives, research methodology, data collection, data analysis, presentation of findings, interpretation of results, ethics approval, and derivation of conclusions. The complete methodological quality appraisal results are presented in Multimedia Appendix 3 [25-27,32-51].

### Themes

Through iterative reading and comparative analysis, four overarching themes and twelve subthemes were synthesized (Textbox 1): (1) patient empowerment and enhanced self-management, (2) sense of security and continuity of care under professional support, (3) variations in acceptance and emotional responses, and (4) barriers and challenges in implementing eHealth interventions. The correspondence between each theme and subtheme and the included studies is presented in Multimedia Appendix 4 [25-27,32-51].

Textbox 1. **Themes and subthemes.**

**Theme 1: patient empowerment and enhanced self-management**

- Improved health literacy and disease awareness
- Strengthened self-efficacy and confidence
- Behavioral change and health improvement

**Theme 2: sense of security and continuity of care under professional support**

- Sense of security from remote monitoring
- Continuity of care and collaborative engagement
- Communication and relationship building

**Theme 3: variations in acceptance and emotional responses**

- Positive acceptance and motivation
- Negative attitudes and psychological resistance
- Individual differences and applicability

**Theme 4: barriers and challenges in implementing eHealth interventions**

- Technological usability and user experience
- Technical issues and use barriers
- Concerns about sustainability

To further clarify the trustworthiness and interpretive value of the synthesized findings, this study used the JBI ConQual approach to assess the level of confidence in the evidence for each synthesized theme and subtheme. The ConQual assessment was based primarily on 2 domains: methodological dependability, which was assessed according to the methodological quality of the corresponding included studies, and credibility of findings, which reflected the extent to which the original participant quotations supported the synthesized findings. Overall, the level of confidence in the evidence was rated as moderate for all 4 synthesized themes and ranged from moderate to high for the 12 subthemes. Downgrading was mainly attributable to limitations in the credibility of some findings, as several synthesized findings were supported by both unequivocal and credible findings rather than solely by unequivocal findings. In addition, although the overall methodological quality of the included studies was acceptable, several studies insufficiently reported researcher reflexivity and the reciprocal influence between the researchers and the research process. The ConQual assessment results for the 4 synthesized themes are presented in Table 2, and the complete results for each synthesized theme and subtheme are provided in Multimedia Appendix 5 [25-27,32-51].

**Table 2.** ConQual^a^ assessment of the synthesized findings.

Synthesized findings	Contributing studies, n	Dependability	Credibility	ConQual rating
1. Patient empowerment and enhanced self-management	9 [25,26,32,37,42,43,45,48,51]	High	Moderate	Moderate
2. Sense of security and continuity of care under professional support	11 [25,32,33,36-38,44,45,47,50,51]	High	Moderate	Moderate
3. Variations in acceptance and emotional responses	8 [27,32,35,36,39-41,49]	High	Moderate	Moderate
4. Barriers and challenges in implementing eHealth interventions	10 [25,34-39,43,46,50]	High	Moderate	Moderate

a ConQual: Confidence in the Evidence from Reviews of Qualitative Research.

### Theme 1: Patient Empowerment and Enhanced Self-Management

#### Subtheme 1: Improved Health Literacy and Disease Awareness

The included studies showed that patients perceived nurse-led eHealth interventions as improving their understanding of CHF-related knowledge, including disease mechanisms, symptom recognition, dietary management, medication use, and responses to abnormal signs or changes in condition. Patients’ accounts indicated that these interventions increased access to disease-related information and deepened their understanding of the practical elements of daily management. This helped them connect abstract disease knowledge with real-life situations and supported subsequent self-management.

... [the programme] is really very good, because I have learnt a lot of things that I did not know last time, like how to read the sodium amount in the food label ...

[Patient] [25]

No, I think the information has been good, simple, and clear.

[Patient] [46]

#### Subtheme 2: Strengthened Self-Efficacy and Confidence

Building on this improved understanding, some patients described a more positive perception of their ability to manage their condition, reflected in a stronger sense of control over their health and greater confidence in self-monitoring and sustained self-management. Rather than seeing themselves solely as passive recipients of care, patients increasingly described daily monitoring, record-keeping, and symptom response as management tasks they could undertake themselves.

I can control my condition more easily because I have more knowledge about how my condition is right now ... I am quite confident ...

[Patient] [25]

It’s good to be able to input data consciously, when done every day.

[Patient] [26]

#### Subtheme 3: Behavioral Change and Health Improvement

Building on improvements in knowledge and self-efficacy, the included studies further showed that patients reported tangible adjustments to their daily health behaviors. These changes were mainly reflected in more consistent dietary control, more proactive monitoring of physiological signs and symptoms, more structured medication management, and greater attention to self-observation and self-regulation in daily life. Some patients also reported symptom relief, improved activity tolerance, and better overall health status. Patients’ accounts indicated that nurse-led eHealth interventions not only influenced their understanding of the disease but were also associated with perceived changes in self-management behaviors and subjective health status.

… the doctors kept saying to me that you can self-medicate with fluid tablets. And I would think “oh no [laugh], I don’t know what I’m doing here, so I’m not going to do that ...” But then the [telemonitoring staff] at the other end said take another fluid tablet ... And then gradually, I started to realise that when I felt unwell I was able to think “oh, you know, take another tablet or half a tablet.”

[Patient] [37]

It felt so safe. I monitored my blood pressure and pulse. In addition, I observed myself and how my body reacted. When I walked the dog, I would watch how my body handled the strain. I have been walking further and further each day.

[Patient] [51]

### Theme 2: Sense of Security and Continuity of Care Under Professional Support

#### Subtheme 1: Sense of Security From Remote Monitoring

Multiple studies indicated that continuous data transmission, remote monitoring, and timely feedback allowed patients to perceive ongoing professional oversight, which was associated with an enhanced sense of security during postdischarge management. This sense of security mainly arose from patients’ confidence that changes in their condition could be identified promptly and addressed through professional responses. For patients, reassurance arose less from the technology itself than from the sustained attention and accessible professional support that the technology represented.

Yes, they are probably keeping an eye on me. If something happens there is somebody on the other end that will see that; aha, something’s happening here.

[Patient] [44]

Without the comments from my nurse navigator and the close monitoring, the remote patient monitoring would not have provided the same sense of security.

[Patient] [45]

I feel secure knowing that people at the hospital are observing my condition through this tool.

[Patient] [47]

#### Subtheme 2: Continuity of Care and Collaborative Engagement

Nurse-led eHealth interventions extended nursing care beyond hospital discharge and were perceived as reducing gaps in care continuity commonly seen in traditional models during the transition from hospital to home. Patients generally perceived that remote follow-up, home visits, and individualized feedback enabled them to report problems continuously, receive symptom assessment, and obtain timely adjustments to interventions during the postdischarge period. At the same time, these interventions facilitated information sharing and collaboration between nurses and physicians, allowing patients to receive more continuous professional support even when outpatient consultation time was limited or access to in-person care was restricted.

I hope that whatever you saw during home visit, [you] can let my doctor know too ... my problems ... because doctor’s consultation is very short, only have 10 minutes ... but during home visit, I can tell you more ...

[Patient] [25]

What bothered me the most was that I couldn’t get rid of the dizziness, so I thought it would be best to contact the out-patient clinic ... but before I could do that the nurse contacted me because she saw I had been reporting dizziness. She discussed it with a supervising doctor and some of my medications were changed. That was really an improvement ... a total change to my everyday life.

[Patient] [51]

#### Subtheme 3: Communication and Relationship Building

Beyond monitoring and feedback functions, patients also placed importance on the mode of communication itself. The studies showed that timely, individualized, and respectful communication was perceived as supporting patients’ sense of trust, acceptance of the intervention, and engagement with it. Patients’ accounts also suggested that, in the context of eHealth, technology did not necessarily weaken nurse-patient interaction. Instead, when communication conveyed understanding, presence, and responsiveness, technology could function as a means of sustaining the nurse-patient relationship.

Yes, yes I do. And I really appreciate that because you took your time to come to my home, be there. It wasn’t a very formal meeting. It’s like talking to friends. So, it is good ... we can just talk, not just about medical advice. We can sit down and talk, and have a good conversation.

[Patient] [25]

Yeah, I would love that. Just to come and make sure I’m alright ... This beats having to wait for an appointment.

[Patient] [50]

### Theme 3: Variations in Acceptance and Emotional Responses

#### Subtheme 1: Positive Acceptance and Motivation

Some patients expressed positive attitudes toward nurse-led eHealth interventions and reported a willingness to participate. Their motivation was mainly related to expectations of health improvement, recognition of the potential benefits of the intervention, and openness to learning new technologies. In addition, encouragement from health care professionals, initial training and support, and the availability of convenient devices or services were perceived as supporting both patients’ initial willingness to try the intervention and their continued engagement. Patients’ accounts suggested that active acceptance was not simply a matter of technological preference but reflected a readiness to engage once patients perceived the potential value of the intervention.

It helped me in the sense that I was weighing myself almost every day, I know my pressure. I have statistical data. If it increases a lot, I try to find out why. Yes, it forces me to be more vigilant.

[Patient] [27]

Yeah, I’d definitely be willing to learn, you know, even at my age, I would still like to learn.

[Patient] [35]

#### Subtheme 2: Negative Attitudes and Psychological Resistance

In contrast to active acceptance, some patients expressed reservations or resistance toward eHealth interventions. The main reasons included unfamiliarity with new technologies, concerns about their learning capacity, doubts about the necessity of the intervention, and discomfort with being monitored or tracked. Such reservations were not solely attributable to limited technical competence; they also involved patients’ understanding of disease management, their evaluation of the value of technology, and their subjective perceptions of digital monitoring. Together, these factors appeared to function as psychological barriers to participation and may contribute to insufficient engagement or premature withdrawal.

I don’t know if it’s useful or not. I think with this illness, I just need to take medication and diuretics. Using some kind of management software might not be of much help.

[Patient] [40]

It feels like being tracked by someone—I find it quite off-putting.

[Patient] [41]

#### Subtheme 3: Individual Differences and Applicability

The findings showed individual variation in patients’ acceptance of eHealth interventions, which was associated with factors such as age, educational level, health literacy, cognitive status, and prior experience with technology. Different forms of eHealth technology also placed different demands on patients’ capacity for active participation, resulting in varied perceptions of suitability. Compared with interventions primarily involving data uploading, remote monitoring, and nurse feedback, app- or web-based interventions generally relied more heavily on patients’ active use, information comprehension, and sustained engagement, making them more susceptible to individual differences in digital competence. By contrast, remote monitoring, structured telephone support, and proactive nurse feedback appeared to reduce the burden of continuous input and complex operational procedures to some extent. Patients’ acceptance was influenced not only by individual characteristics but also by the participation requirements of specific technological modalities and the intensity of nursing support.

Sigh! I only went to school up to the third grade. How could I possibly keep using this kind of high-tech app? I just can’t stick with it—I don’t know how to use it.

[Patient] [40]

Yeah, but, it’s been like ... I’ve gotten help with that information too, you know. I don’t understand all the words, so that’s like ... my wife has to help me with that. She is everything, you know. Yeah, that’s ... I have trouble coming up with any examples, to be honest (laughing). It might be something that I don’t really understand, that she can understand better, and then she’ll explain it to me.

[Patient] [49]

### Theme 4: Barriers and Challenges in Implementing eHealth Interventions

#### Subtheme 1: Technological Usability and User Experience

Usability was a key prerequisite for patients’ positive experiences with eHealth interventions and their sustained engagement. The included studies showed that patients perceived interventions with simple interfaces, clear operational steps, and flexible timing as easier to accept and more supportive of confidence in continued use. For patients with CHF, particularly older adults, a positive user experience was mainly reflected in low cognitive burden, ease of use, and the feasibility of sustained use over time. Patients’ accounts indicated that usability was not merely an additional feature but a central factor shaping their willingness to initiate and continue participation in the intervention.

I think the best is simple and useful ... yes ... because we need to work, if it is too complex, we feel very troublesome. So, I think the simpler the better ... And also, we are not that young now, sometimes we cannot remember so many things.

[Patient] [25]

If I wanted to sit down and do it at two in the night or five in the morning or in the middle of the day then this was fine I could choose when to carry out my exercises [...] it is an advantage to be able to do it at a time of my choice.

[Patient] [43]

#### Subtheme 2: Technical Issues and Use Barriers

Although patients generally acknowledged the convenience of eHealth interventions in facilitating information access, remote communication, and self-monitoring, the barriers they encountered varied across intervention types. App- and web-based interventions were more often associated with operational difficulties, such as login failures, system errors, complex interfaces, substantial information-processing demands, and inconvenient data entry. These problems were particularly prominent among older patients and those with visual impairment or limited digital experience. By contrast, the main barriers associated with telemonitoring interventions did not always arise from complex interfaces but were more often related to device stability, data transmission, timeliness of feedback, and technical support. When patients were unable to complete data uploading successfully or could not obtain timely assistance when problems occurred, remote services originally intended to provide reassurance could instead be experienced as frustrating and undermine trust. Therefore, the technical barriers experienced by patients were not uniform but varied according to the specific technological modality and its interactive demands.

... when I can’t get it to connect it gets me very frustrated ...

[Patient] [32]

I’m old. I can’t see well, I can’t read much, I can’t type, and it’s too difficult to use (mHealth).

[Patient] [39]

#### Subtheme 3: Concerns About Sustainability

Beyond short-term user experience, patients also expressed concerns about the sustainability of eHealth interventions. These concerns mainly involved the cost of devices or services, the possibility of future charges, the availability of ongoing technical support, and the credibility of information provided by the platform. Patients’ accounts indicated that integration of eHealth interventions into disease management depended not only on immediate usability but also on patients’ ongoing appraisal of financial burden, sustained support, and information reliability. Such concerns shaped patients’ evaluations of the long-term value of eHealth interventions.

Because most of the people who would be in this situation have had problems normally at their age when they are on a fixed income or retirement or something. So it depends, unless a person is really wealthy. Because it used to be paycheck to paycheck, and now you’re in retirement and now you have to take care of additional medical bills, this extra addition of expenditure could be a factor.

[Patient] [35]

I think we need something to tell us that hey you won’t be charged later, and if you are you get a refund or something.

[Patient] [50]

## Discussion

### Principal Findings

This qualitative systematic review and meta-synthesis integrated patients’ experiences of nurse-led eHealth interventions for CHF. The findings were reflected in 4 main aspects. Nurse-led eHealth interventions were perceived as supporting disease-related knowledge, self-efficacy, and adjustments in daily self-management behaviors; continuous monitoring, remote follow-up, and timely feedback were associated with patients’ sense of security and perceived continuity of care; willingness to engage varied across individuals and technological modalities; and usability, technical barriers, and concerns about long-term sustainability remained important constraints during implementation. These findings indicate that patients’ experiences encompassed not only empowerment and support but also technological adaptation and implementation burden.

This review first showed that nurse-led eHealth interventions were perceived as supporting patients’ understanding of CHF and its daily management. Patients not only received more information on symptom recognition, dietary control, medication use, and responses to abnormal signs or changes in condition but also gradually translated this information into practical knowledge relevant to their own life contexts [25,26,48,51]. This suggests that the value of such interventions lies not merely in information provision but also in helping patients transform disease-related knowledge into understandable and applicable management experience. Previous studies have emphasized the foundational role of health education in chronic disease management [52,53], and this review further shows, from the perspective of patients’ experience, that nurse-led eHealth interventions may improve both the comprehensibility and practical usefulness of disease-related information.

On this basis, some patients developed a more positive perception of their disease management capacity, as reflected in a stronger sense of control over their health condition and greater confidence in self-monitoring and sustained management [25,26,48]. This finding indicates that the experiential value of nurse-led eHealth interventions extends beyond supporting patients’ understanding of disease-related information to helping them gradually develop trust in their own management capacity. Previous chronic disease management research has identified self-efficacy as an important factor influencing long-term engagement and sustained self-management [54,55]. The findings of this review are broadly consistent with this view, suggesting that whether patients can incorporate these interventions into daily life depends not only on whether they know how to manage their condition but also on whether they believe they are capable of implementing these behaviors. Patients reported greater regularity and initiative in dietary control, symptom and sign monitoring, medication management, and daily activity planning [25,37], and some perceived symptom relief, improved activity tolerance, and better overall condition [43,44,51]. However, because this review focused on patients’ subjective experiences rather than intervention effectiveness, these findings should be interpreted as perceived changes rather than evidence of objective clinical outcomes.

Beyond empowerment, another important finding was patients’ emphasis on a sense of security and continuity of care. Patients repeatedly reported that continuous data transmission, remote monitoring, and timely feedback gave them a sense of ongoing professional oversight after discharge and were perceived as reducing uncertainty about changes in their condition [33,34,47]. This suggests that patients’ sense of security arose less from the technology itself than from the perceived presence of continued professional support [56]. Previous studies on remote care have similarly shown that patients value technology not because it is advanced, but because it can reduce the perceived distance between patients and professional support [57,58]. The present findings further support this view.

At the same time, nurse-led remote follow-up, home visits, and individualized feedback were perceived as extending traditional care across time and space [37,38,42]. Compared with brief outpatient encounters with limited opportunities to express concerns [25,56,59], these interventions were perceived as providing patients with ongoing opportunities to report problems, receive symptom assessment, and obtain timely recommendations. The continuity perceived by patients was reflected not only in uninterrupted service but also in the continuous receipt, interpretation, and response to patient information. In addition, this review showed that technological involvement did not necessarily weaken the nurse-patient relationship; under certain conditions, it could support trust and relational continuity [47,50]. Patients placed particular value on timely, individualized, and respectful communication, viewing it as a key basis for accepting and sustaining engagement with the intervention [60]. This suggests that, in the eHealth context, technology does not necessarily replace the nurse-patient relationship but can extend it into the postdischarge setting by enabling ongoing communication and professional responsiveness [61].

Regarding willingness to accept the intervention, this study showed variation in patients’ attitudes toward nurse-led eHealth interventions. Some patients perceived these interventions as beneficial support and were willing to learn and remain engaged [27,35,36], whereas others expressed reservations or resistance [35,39-41]. This variation was not determined solely by patients’ ability to use technology but was associated with multiple factors, including age, educational level, health literacy, cognitive status, prior technological experience, and understanding of disease management approaches [59]. These findings indicate that patients’ acceptance of eHealth interventions is a process shaped by cognitive, emotional, and contextual attributes rather than merely a matter of technology adoption [62,63]. This result is broadly consistent with existing digital health research showing that acceptance is influenced by multiple factors while further revealing how such variation is manifested among people with CHF from the perspective of patient experience.

This study further showed that different forms of eHealth technology served distinct nursing functions and were associated with differences in patient experience [35,40,41,49]. Apps and web-based platforms relied more heavily on patients’ active reading, recording, and feedback, and were therefore more likely to reveal differences in learning ability, operational competence, and sustained engagement. Telemonitoring primarily supported patients’ perception that their condition was being attended to through data uploading and professional feedback, with its value mainly reflected in a sense of security and the experience of continuous monitoring. Telephone and video follow-up, as well as home-based support, were more closely aligned with an extension of conventional nursing relationships, from which patients mainly obtained convenient communication, individualized explanations, and relational continuity [25,33,36,50,64,65]. These findings suggest that patients’ acceptance or resistance should be understood in relation to the specific technological modality. The design of nurse-led eHealth interventions should not assume a single uniform model but should instead align patients’ capabilities and support needs with the nursing functions performed by different technological modalities.

This review also revealed multiple barriers during implementation. Usability was closely related to patients’ willingness to initiate and sustain engagement [25,34,35,43]. Patients repeatedly referred to interface simplicity, clarity of procedural steps, ease of remembering how to operate the system, and flexibility in timing [25,43], indicating that for patients with CHF, particularly older adults, usability is a key prerequisite for effective implementation and sustained use [66]. Technical problems also varied across intervention forms. App- and web-based platforms were more likely to involve login difficulties, challenges in understanding information, and frustration caused by interface complexity [32,34,39,43,67], whereas remote monitoring interventions more often involved problems related to device functioning, data transmission, timely feedback, and technical support [32,44,45,47,68]. Beyond these immediate challenges, patients also expressed concerns about long-term sustainability [69]. Cost, the possibility of future charges, the availability of ongoing technical support, and the credibility of information sources all shaped patients’ judgments about the long-term value of eHealth interventions [70,71]. These findings suggest that eHealth interventions should focus not only on initial acceptance but also on long-term affordability, sustained support, and the maintenance of trust.

### Limitations

This review has several limitations. First, although a systematic search of multiple databases and gray literature was conducted, the included studies were restricted to published papers in Chinese and English, which may have introduced publication and language bias and affected the comprehensiveness of the evidence. Second, the participants in the included studies were predominantly patients with CHF who had basic cognitive and communication abilities; therefore, the representation of the oldest-old population, patients with severe cognitive impairment, or patients with strong resistance to technology was limited, which may constrain the applicability of the findings to these specific populations. Third, most of the included studies were conducted in high- and middle-income countries or regions, particularly in Europe, North America, and East Asia, while evidence from lower-income regions and culturally diverse contexts remained relatively scarce, which may limit the cross-cultural transferability of the findings to some extent. Fourth, the forms of eHealth intervention and their application contexts varied across studies. Although this review identified shared experiential patterns through thematic synthesis, subtle differences across intervention modalities may have been attenuated during the process of integration. Fifth, some primary studies showed limitations in reporting their philosophical stance, researcher positioning, and reflexivity, which may have affected the transparency of interpretation in the primary findings and, in turn, the robustness of the synthesized evidence. Finally, this review focused on patients’ experiences rather than intervention effectiveness. Therefore, the findings should be interpreted as reflecting how patients perceived and interpreted nurse-led eHealth interventions and should not be directly extrapolated to clinical or patient-reported outcomes such as rehospitalization, disease progression, or quality of life.

### Implications for Clinical Practice and Research

This review suggests that nurse-led eHealth interventions may have practical value in CHF management, but their implementation requires attention to patient characteristics, technological suitability, and nursing system support. For clinical practice, intervention tools should be designed with simple and user-friendly interfaces, adequate training, and individualized adjustments to reduce barriers for older patients and support sustained engagement. In remote care delivery, nurses should emphasize sustained supportive presence and individualized responsiveness. Relatively consistent follow-up teams, proactive feedback, and respectful communication may support patients’ trust and their perception of continuity of care. At the organizational level, adequate staffing, training, and institutional support should be provided. Nurses’ responsibilities in data monitoring, symptom feedback, health education, and psychological support should be clearly defined, and mechanisms for interdisciplinary collaboration and data security should be strengthened to support the sustainable operation of nurse-led models.

Future research should further clarify the fit among different technological modalities, patient characteristics, and care contexts, with particular attention to adaptation strategies for the oldest-old population, individuals with low digital literacy, and patients with multimorbidity. In addition, future studies could build on qualitative research by adopting mixed methods designs to examine the relationship between patients’ subjective experiences and objective clinical indicators. For example, patient-reported measures such as technology acceptability, use burden, self-efficacy, or satisfaction could be analyzed alongside readmission, emergency department visits, mortality, and adherence-related outcomes. Such work could provide a more comprehensive understanding of the role of patient experience in shaping both intervention implementation and clinical outcomes. Such research could help move beyond purely descriptive accounts of experience or isolated evaluations of effectiveness and support the optimization and application of patient-centered, nurse-led eHealth interventions.

### Conclusions

This review systematically synthesized the experiences of patients with CHF receiving nurse-led eHealth interventions from the patient perspective. The findings showed that such interventions could enhance disease understanding, strengthen self-efficacy, support adjustments in daily self-management behaviors, and improve patients’ sense of security and perceived continuity of care in postdischarge settings. At the same time, patients’ acceptance and sustained engagement were jointly influenced by individual characteristics, technological modalities, usability barriers, and concerns about long-term sustainability. These findings indicate that the significance of nurse-led eHealth interventions lies not only in extending the temporal and spatial reach of nursing services but also in maintaining patients’ perceived access to professional support, timely responses, and the nurse-patient relationship during out-of-hospital management. Future practice and research should place greater emphasis on the heterogeneity of patient experiences and develop more appropriate matching pathways between different technological modalities and patient characteristics, thereby improving the adaptability and sustainability of nurse-led eHealth interventions in CHF management.

## Supplementary material

10.2196/82714 Multimedia Appendix 1 Search strategies for all databases.

10.2196/82714 Multimedia Appendix 2 Characteristics of the included studies.

10.2196/82714 Multimedia Appendix 3 Results of the critical appraisal of the studies included.

10.2196/82714 Multimedia Appendix 4 Mapping of themes and subthemes to included studies.

10.2196/82714 Multimedia Appendix 5 Full Confidence in the Evidence from Reviews of Qualitative Research assessment of the synthesized findings.

10.2196/82714 Checklist 1 PRISMA checklist.

## Abbreviations

CHF: chronic heart failure
ConQual: Confidence in the Evidence from Reviews of Qualitative Research
JBI: Joanna Briggs Institute
PRISMA: Preferred Reporting Items for Systematic Reviews and Meta-Analyses
PRISMA-S: Preferred Reporting Items for Systematic Reviews and Meta-Analyses Literature Search Extension
