# Supplementary material for: Patients’ Experiences of Nurse-Led eHealth Interventions for Chronic Heart Failure: Qualitative Systematic Review and Meta-Synthesis
Source: J Med Internet Res. 2026 Jul 6;28:e82714. doi: 10.2196/82714 (PMC13335749; doi:10.2196/82714)
Supplement: Multimedia Appendix 1 [file jmir-v28-e82714-s001.docx]

**Multimedia Appendix 1.** Search Strategies for All Databases

**PubMed**

("nurse-led" OR "nurse managed" OR "nurse-directed" OR "nurse-delivered" OR nurs*) AND ("heart failure" OR "cardiac failure" OR "myocardial failure" OR "congestive heart failure" OR "left-sided heart failure" OR "right-sided heart failure" OR "heart decompensation") AND (telemedicine OR telehealth OR "telecare" OR "telemonitor" OR "telecoaching" OR "telenursing" OR "ehealth" OR "e-health" OR "mhealth" OR "m-health" OR "gerontechnology" OR "telerehabilitation" OR "e-rehabilitation" OR "telecommunication" OR videoconferenc* OR teleconferenc* OR internet OR computer* OR mobile OR phone* OR smartphone* OR telephone* OR tablet* OR email OR "e-mail" OR SMS OR apps OR applications OR "social media" OR wireless OR virtual OR remote OR distant OR technolog*) AND (qualitative OR "focus group*" OR interview* OR experienc* OR attitud* OR feel* OR respons* OR perspectiv* OR opin* OR phenomenolog* OR "lived experience*" OR narrative* OR ethnograph* OR "grounded theory" OR "content analysis")

**Web of Science**

"nurse-led" OR "nurse managed" OR "nurse-directed" OR "nurse-delivered" OR nurs* (All Fields) and "heart failure" OR "cardiac failure" OR "myocardial failure" OR "congestive heart failure" OR "left-sided heart failure" OR "right-sided heart failure" OR "heart decompensation" (All Fields) and telemedicine OR telehealth OR telecare OR telemonitor* OR teleteaching OR telenursing OR "tele-nursing" OR eHealth OR "e-health" OR mHealth OR "m-health" OR gerontechnology OR telerehabilitation OR "e-rehabilitation" OR telecommunication* OR videoconferenc* OR teleconferenc* OR internet* OR computer* OR mobile* OR phone* OR smartphone* OR telephone* OR tablet* OR email* OR "e-mail*" OR SMS OR app OR apps OR application* OR "social media" OR wireless* OR virtual* OR remote* OR distant* OR technolog* (All Fields) and qualitative OR "focus group*" OR interview* OR experienc* OR attitud* OR feel* OR respons* OR perspectiv* OR opin* OR phenomenolog* OR "lived experience*" OR narrative* OR ethnograph* OR "grounded theory" OR "content analysis" (All Fields)

**Cochrane Library**

"nurse-led" OR "nurse managed" OR "nurse-directed" OR "nurse-delivered" OR nurs* in All Text AND "heart failure" OR "cardiac failure" OR "myocardial failure" OR "congestive heart failure" OR "left-sided heart failure" OR "right-sided heart failure" OR "heart decompensation" in All Text AND telemedicine OR telehealth OR telecare OR telemonitor* OR telecoaching OR telenursing OR "tele-nursing" OR eHealth OR "e-health" OR mHealth OR "m-health" OR gerontechnology OR telerehabilitation OR "e-rehabilitation" OR telecommunication* OR videoconferenc* OR teleconferenc* OR internet* OR computer* OR mobile* OR phone* OR smartphone* OR telephone* OR tablet* OR email* OR "e-mail*" OR SMS* OR app* OR application* OR "social media" OR wireless* OR virtual* OR remote* OR distant* OR technolog* in All Text AND qualitative OR "focus group*" OR interview* OR experienc* OR attitud* OR feel* OR respons* OR perspectiv* OR opin* OR phenomenolog* OR "lived experience*" OR narrative* OR ethnograph* OR "grounded theory" OR "content analysis" in All Text - (Word variations have been searched)

**Embase**

('nurse-led' OR 'nurse managed' OR 'nurse-directed' OR 'nurse-delivered' OR nurs*) AND ('heart failure' OR 'cardiac failure' OR 'myocardial failure' OR 'congestive heart failure' OR 'left-sided heart failure' OR 'right-sided heart failure' OR 'heart decompensation') AND (telemedicine OR telehealth OR 'telecare' OR 'telemonitor' OR 'telecoaching' OR 'telenursing' OR 'ehealth' OR 'e-health' OR 'mhealth' OR 'm-health' OR 'gerontechnology' OR 'telerehabilitation' OR 'e-rehabilitation' OR 'telecommunication' OR videoconferenc* OR teleconferenc* OR internet OR computer* OR mobile OR phone* OR smartphone* OR telephone* OR tablet* OR email OR 'e-mail' OR sms OR apps OR applications OR 'social media' OR wireless OR virtual OR remote OR distant OR technolog*) AND (qualitative OR 'focus group*' OR interview* OR experienc* OR attitud* OR feel* OR respons* OR perspectiv* OR opin* OR phenomenolog* OR 'lived experience*' OR narrative* OR ethnograph* OR 'grounded theory' OR 'content analysis')

**CINAHL**

("nurse-led" OR "nurse managed" OR "nurse-directed" OR "nurse-delivered" OR nurs*) AND SU ("heart failure" OR "cardiac failure" OR "myocardial failure" OR "congestive heart failure" OR "left-sided heart failure" OR "right-sided heart failure" OR "heart decompensation") AND (telemedicine OR telehealth OR telecare OR telemonitor* OR telecoaching OR telenursing OR "tele-nursing" OR eHealth OR "e-health" OR mHealth OR "m-health" OR gerontechnology OR telerehabilitation OR "e-rehabilitation" OR telecommunication* OR videoconferenc* OR teleconferenc* OR internet* OR computer* OR mobile* OR phone* OR smartphone* OR telephone* OR tablet* OR email* OR "e-mail*" OR SMS* OR app* OR application* OR "social media" OR wireless* OR virtual* OR remote* OR distant* OR technolog*) AND (qualitative OR "focus group*" OR interview* OR experienc* OR attitud* OR feel* OR respons* OR perspectiv* OR opin* OR phenomenolog* OR "lived experience*" OR narrative* OR ethnograph* OR "grounded theory" OR "content analysis")

**CNKI**

全文 = (护士主导 OR 护士管理 OR 护士指导 OR 护士实施 OR 护士 OR 护理) AND 主题 = (心力衰竭 OR 心衰 OR 心功能不全 OR 充血性心力衰竭 OR 左心衰竭 OR 右心衰竭 OR 心功能代偿不全) AND 全文 = (远程医疗 OR 远程健康 OR 远程护理 OR 远程监测 OR 远程指导 OR 电子健康 OR 移动健康 OR 老年科技 OR 远程康复 OR 电子康复 OR 电信通信 OR 视频会议 OR 网络会议 OR 互联网 OR 计算机 OR 移动 OR 手机 OR 智能手机 OR 电话 OR 平板 OR 电子邮件 OR 短信 OR 应用程序 OR 社交媒体 OR 无线 OR 虚拟 OR 远程 OR 技术) AND 全文 = (质性 OR 焦点小组 OR 访谈 OR 经验 OR 态度 OR 感受 OR 反应 OR 观点 OR 视角 OR 意见 OR 现象学 OR 生活经验 OR 叙事 OR 人种学 OR 扎根理论 OR 内容分析)

**WanFang**

全部:(护士主导 OR 护士管理 OR 护士指导 OR 护士实施 OR 护士 OR 护理) AND 主题:(心力衰竭 OR 心衰 OR 心功能不全 OR 充血性心力衰竭 OR 左心衰竭 OR 右心衰竭 OR 心功能代偿不全) AND 全部:(远程医疗 OR 远程健康 OR 远程护理 OR 远程监测 OR 远程指导 OR 电子健康 OR 移动健康 OR 老年科技 OR 远程康复 OR 电子康复 OR 电信通信 OR 视频会议 OR 网络会议 OR 互联网 OR 计算机 OR 移动 OR 手机 OR 智能手机 OR 电话 OR 平板 OR 电子邮件 OR 短信 OR 应用程序 OR 社交媒体 OR 无线 OR 虚拟 OR 远程 OR 技术) AND 全部:(质性 OR 焦点小组 OR 访谈 OR 经验 OR 态度 OR 感受 OR 反应 OR 观点 OR 视角 OR 意见 OR 现象学 OR 生活经验 OR 叙事 OR 人种学 OR 扎根理论 OR 内容分析)

**VIP**

任意字段:(护士主导 OR 护士管理 OR 护士指导 OR 护士实施 OR 护士 OR 护理) AND 提名或关键词:(心力衰竭 OR 心衰 OR 心功能不全 OR 充血性心力衰竭 OR 左心衰竭 OR 右心衰竭 OR 心功能代偿不全) AND 任意字段(远程医疗 OR 远程健康 OR 远程护理 OR 远程监测 OR 远程指导 OR 电子健康 OR 移动健康 OR 老年科技 OR 远程康复 OR 电子康复 OR 电信通信 OR 视频会议 OR 网络会议 OR 互联网 OR 计算机 OR 移动 OR 手机 OR 智能手机 OR 电话 OR 平板 OR 电子邮件 OR 短信 OR 应用程序 OR 社交媒体 OR 无线 OR 虚拟 OR 远程 OR 技术) AND 任意字段:(质性 OR 焦点小组 OR 访谈 OR 经验 OR 态度 OR 感受 OR 反应 OR 观点 OR 视角 OR 意见 OR 现象学 OR 生活经验 OR 叙事 OR 人种学 OR 扎根理论 OR 内容分析)
