# Supplementary material for: Patients’ Experiences of Nurse-Led eHealth Interventions for Chronic Heart Failure: Qualitative Systematic Review and Meta-Synthesis
Source: J Med Internet Res. 2026 Jul 6;28:e82714. doi: 10.2196/82714 (PMC13335749; doi:10.2196/82714)
Supplement: Multimedia Appendix 2 [file jmir-v28-e82714-s002.docx]

**Multimedia Appendix 2.** Characteristics of the Included Studies

| Author, year | Country | Setting | Sample | Study design | Aim | Data collection method | Data collection mode | Duration of the interview (min) | Data analysis | Finding (including themes) |
| --- | --- | --- | --- | --- | --- | --- | --- | --- | --- | --- |
| Auton et al, 2023 [32] | UK | In regional heart failure service | 79 HF patients :  51 males  28 females | Mixed method design | To (1) evaluate how patients and nurses use this type of RM (usage type), (2) evaluate patients’ and nurses’ user feedback on this type of RM (user experience), and (3) directly compare the usage type and user experience of patients and nurses using the same type of RM platform at the same time. | Semi-structured interview | The focus group | Unknown | Thematic analysis | 1.Positive impacts  2.Negative impacts |
| Birkhoff et al, 2021 [33] | USA | In their home or assisted living residence | 34 HF patients :  16 males  18 females | Mixed method design | To explore heart failure patients’ abilities, experience, and satisfaction to use and adopt a virtual nurse visit. | Semi-structured interview | Unknown | 20-30 min | Thematic analysis | 1. Perceived safety during COVID-19 2. Preferences for delivery of care 3. User experiences and challenges with the VNV service, 4. Satisfaction with VNV |
| Buck et al, 2017 [34] | USA | In hospital | 12 HF patients | A qualitative research study | To present the qualitative analysis of participant post-intervention interviews from the tablet-delivered Penn State Heart Assistant intervention. | Semi-structured interview | Face-to-Face interviews | An average interview lasting 14 min | Thematic analysis | 1. Benefits: information sharing with others, usability and learnability, use of help resources   2. Suggestions: continuing use after the study, technical problems, participant suggested improvements. |
| Author, year | Country | Setting | Sample | Study design | Aim | Data collection method | Data collection mode | Duration of the interview (min) | Data analysis | Finding (including themes) |
| Cajita et al, 2018 [35] | USA | In hospital | 10 HF patients | A qualitative research study | To assess the perceptions of older adults with heart failure regarding the use of mobile technology and to identify potential facilitators of and barriers to mHealth adoption | Semi-structured interview | Face-to-Face interviews | Interviews lasted from 25 to 45 minutes | Thematic analysis | 1. facilitators: previous experience with mobile technology, willingness to learn mHealth, ease of use, presence of useful features, adequate training, free equipment, and doctor’s recommendation;   2. barriers: lack of knowledge regarding how to use mHealth, decreased sensory perception, lack of need for technology, poorly designed interface, cost of technology, and limited/fixed income |
| Carter et al, 2022 [36] | USA | In the community | 27 HF patients | A qualitative research study | To explore patient perceptions on HF management at home, the use of home-based remote monitoring, and the value of home-based care | Semi-structured interview | Telephone interview | 30-45min | Thematic analysis | 1. home-based care plan instructions are understood;   2. following medication, diet, and fluid management instructions are challenging due to difficult adherence to and implementation at home; 3. financial limitations serve as barriers to acquiring healthy food;  4. home-based support is a valuable component of managing medications, diet, and fluid;  5. despite limited use of technology, strong willingness to use remote monitoring is present amongst most |
| Author, year | Country | Setting | Sample | Study design | Aim | Data collection method | Data collection mode | Duration of the interview (min) | Data analysis | Finding (including themes) |
| Fairbrother et al,2014  [37] | Lithuania | In their home | 18 HF patients :  12 males  6 females  5professionals | A qualitative research study | To understand the views of patients and professionals on the acceptability and perceived usefulness of telemonitoring in the management of chronic heart failure in the context of day-to-day care provision. | Semi-structured interview | Telephone or face-to-face interview | The average interview duration was 30 minutes | Thematic analysis | 1. Information, support and reassurance   2. Compliance and dependence  3. Changes and challenges  4. Determining the criteria for patient applicability to telemonitoring  5. Continuity of care |
| Gordon et al, 2020 [38] | CAN | In hospital | 14 HF patients  5professionals | Mixed method design | To evaluate the feasibility and patients’ perceived usefulness of a multi-condition Telemonitoring (TM) platform in a nurse-led model of care. | Semi-structured interview | Interviews were conducted onsite or over the telephone | lasting between 30 and 60 min. | Thematic analysis | 1. Making sense of the purpose of TM   2. Engaging and investing in TM  3. Implementing and adopting TM  4. Perceived usefulness and the perceived benefits of TM in CCCs |
| Jiang et al, 2021 [25] | SG | In hospital | 11 HF patients:  7 males  4 females | A descriptive  qualitative research study | To explore participants’ perspectives on a nurse-led, home-based heart failure self-management program (HOM-HEMP) in a randomized controlled trial conducted in Singapore to gain insight into the effectiveness of the study intervention. | Semi-structured interview | Face-to-Face interviews | Unknown | Thematic analysis | 1.Manageability of the intervention  2. Areas for improvement  3. Benefits of visiting  4. Personal accountability in self-care  5. Empowered with knowledge and skills in self-care after the intervention  6.Increase self-efficacy in cardiac care |
| Author, year | Country | Setting | Sample | Study design | Aim | Data collection method | Data collection mode | Duration of the interview (min) | Data analysis | Finding (including themes) |
| Jin et al, 2024 [39] | China | In hospital | 14 HF patients：  9 males  5 females | A descriptive  qualitative research study | To explore the experiences with mHealth use among patients with chronic heart failure and their informal caregivers from a dichotomous perspective. | Semi-structured interview | Face-to-Face interviews | Interviews lasted between 21 minutes and 42 minutes. | Thematic analysis | 1. Opposing experiences with mHealth as human interaction or trauma (great experience with mHealth use; trauma),   2. Supplement instead of replacement (it is useful but better as a reference; offline is unavoidable sometimes),  3. Both agreement and disagreement over who should be the adopter of mHealth (achieving consensus regarding who should adopt mHealth; conflict occurs when considering patients as the adopter of mHealth),  4. For better mHealth (applying mHealth with caution; suggestions for improved mHealth). |
| Lan et al, 2021 [40] | China | In hospital | 11 HF patients：  7 males  4 females | A qualitative research study | To explore the real feelings of patients with low willingness to accept heart failure mHealth applications (apps) and the reasons for abandoning the use of , and to provide a basis for further improvement of heart failure mHealth apps and improvement of intervention strategies. | semi-structured interview | Face-to-Face interviews | 20-40min | Thematic analysis | 1. Socio-demographic characteristics affect willingness to use   2. Individual innovative characteristics affect willingness to use  3. Poor perceived ease of use affects willingness to use  4. Limited perceived usefulness affects willingness to use  5. Presence of negative psychological experiences affects willingness to use |
| Author, year | Country | Setting | Sample | Study design | Aim | Data collection method | Data collection mode | Duration of the interview (min) | Data analysis | Finding (including themes) |
| Liu et al, 2025 [41] | China | In hospital | 17 HF patients：  10 males  7 females | A qualitative research study | To explain the desire and demand for cardiac rehabilitation Apps in heart failure patients,  examine the possible influencing variables ,and give a reference for the promotion of cardiac rehabilitation Apps and service quality improvement. | Semi-structured interview | Face-to-Face interviews | 20-40min | Thematic analysis | 1.Differences in patient attitudes (expectation of mobile health guidance, emphasis on the role of self-regulation, passive reliance on offline treatment)  2. Shifts in health information needs (enhancement of information accuracy, enhancement of information user-friendliness, optimization of information professionalism)  3. Internal characteristics of the App embodied and functional settings (App characteristic attributes, App service functions)  4. Influencing factors in the information environment (service experience experience, recommender identity, parallel support factors) |
| Longhini et al, 2023 [42] | Italy | In a community | 19 HF patients  8 caregivers | Mixed method design | To determine whether a nurse-led care model with telemonitoring in primary care for patients with stable heart failure and their caregivers is feasible and acceptable. | Semi-structured interview | Telephone or face-to-face interview | The interviews lasted from 5 to 30 minutes | Thematic analysis | 1.Input as Adhering to the Model of Care  2. Processes  3. outcomes  4. Perceiving Satisfaction on the Model of Care |
| Author, year | Country | Setting | Sample | Study design | Aim | Data collection method | Data collection mode | Duration of the interview (min) | Data analysis | Finding (including themes) |
| Lundgren et al, 2018 [43] | Sweden | Unknown | 13 HF and depressive symptoms  patients:  9 males  4 females | A qualitative research study | To explore and describe the experiences of participating and receiving health care through a wCBT intervention among persons with heart failure and depressive symptoms. | Semi-structured interview | Telephone interviews | The interviews lasted between 36-72 minutes (median 50 minutes) | Thematic analysis | 1. Something other than usual health care   2. Relevance and recognition 3. Flexible, understandable, and safe  4. Technical problems  5. Improvements by real-time contact  6. Managing my life better |
| Lyngå et al, 2013 [44] | Sweden | In the patient’s home or in a private room at the hospital | 20 HF patients :  15 males  5 females | An explorative and descriptive design with a phenomenographic approach was used | To explore and describe patients’ perceptions of transmission of body weight (BW) and TM, regularly accomplished from patients’ homes to an HF clinic. | Semi-structured interview | Telephone or face-to-face interview | Lasted between 13 and 45 min, | A  phenomenographic approach | 1. The habitual patient   2. The concerned patient  3. The technical patient  4. The secure patient  5. The self-caring patient |
| Østrem et al, 2025 [45] | Norway | In the patient’s home or in a private room at the hospital | 12 HF patients :  9 males  3 females | A qualitative longitudinal approach | To explored short- and long-term experiences of self-care among individuals with heart failure after a six-week, nurse-assisted remote patient monitoring intervention. | Semi-structured interview | Telephone or face-to-face interview | 14-150  minutes | Thematic analysis | At time one:   1. guidance to interpret symptoms and bodily signs; (2) establishing a daily routine in monitoring vital signs;   (3) support for changes in lifestyle and medication adherence;  (4)sense of security.  At time two:   1. increased confidence in bodily awareness and symptom monitoring; 2. recognition of self-care routines; 3. feeling in control due to prior feedback from a previous nurse navigator. |
| Author, year | Country | Setting | Sample | Study design | Aim | Data collection method | Data collection mode | Duration of the interview (min) | Data analysis | Finding (including themes) |
| Säfström et al, 2026 [46] | Sweden | In the patient’s home | 11 HF patients :  6 males  5 females | A feasibility study including interviews with patients and nurses. | To investigate patients and heart failure nurses’ perception of the feasibility of an online support program “Living with Heart Failure”. | Semi-structured interview | Telephone | 6-14  minutes | Thematic analysis | 1.Acceptability  2.Demand  3.Implementation  4.Practicality  5.Integration |
| Sano et al, 2022 [47] | Japan | In clinic | 9 HF patients:  7 males  2 females | A qualitative research study | To clarify patients’ experiences in using the TM tool. | Semi-structured interview | Face-to-Face interview | Approximately 30- to 60-min | Thematic analysis | 1. Habituation of self-care behaviour,   2. No burden for use,  3. A feeling of security,  4. Additional functions  5. Advice rather than instructions. |
| Schmaderer et al, 2021 [26] | USA | In a community | 10 HF patients:  6 males  4 females | A qualitative descriptive research study | To explore the experience of using a self-management mHealth intervention in individuals with heart failure to inform a future mHealth intervention study. | Semi-structured interview | Face-to-Face interview | Approximately 30- to 60-min | Thematic analysis | 1. I didn’t realize, and now I know;   2. It feels good to focus on my health;  3. I am the leader of my health care team; and (4) My health is improving. |
| Son et al, 2020 [48] | Korea | In the patients’ homes or at an outpatient clinic in a hospital | 20 HF patients:  12 males  8 females | A qualitative research study | To explore heart failure patients’ needs and perspectives for using mobile health technology at home before developing a mobile phone-based heart failure self-care intervention. | Semi-structured interviews | Face-to-face interview | Approximately 100 minutes | Thematic analysis | 1.The demand for reliable and customized health information  2. Valuable features of mobile phone applications  3. Barriers to adopting mobile health service  4. Expected benefits of using mobile health technology |
| Author, year | Country | Setting | Sample | Study design | Aim | Data collection method | Data collection mode | Duration of the interview (min) | Data analysis | Finding (including themes) |
| Strandberg et al, 2023 [49] | Sweden | In hospital | 20 HF patients:  16 males  4 females  4 informal carers | A descriptive qualitative study | To describe experiences of self-care management at home when living with hypertension or heart failure, with support from primary care through telemonitoring. | Semi-structured interview | In-person or telephone interviews | Approximately 30-75 minutes | Thematic analysis | 1. Developing the capability to perform self-care with technology as both an intruder and an invited guest |
| Vo et al, 2024 [27] | Canada | In their home | 12 HF patients:  9 males  3 females | A qualitative research study | To explore patients’ experiences using an app for home remote monitoring in heart failure | Semi-structured interview | Face-to-face interview | Unknown | Thematic analysis | 1. Motivation for using the app   2. Benefits of use  3. Experience using the app  4. Suggestions |
| Wali et al, 2020 [50] | Canada | In hospital | 14 HF patients:  7 males  7 females | A qualitative research study | To better understand the self-care challenges that older patients with HF and their informal care providers (CPs) face on a daily basis, leading to the conversion of the SDDST into a user-centered mHealth app. | Semi-structured interview | Face-to-face interview | Approximately 2 hours | Thematic analysis | 1. usability of technology, communication,   2. app customization,  3. complexity of self-care,  4. usefulness of HF-related information,  5. long-term use and cost. |
| Wathne et al, 2025 [51] | Norway | In hospital | 17 HF patients  10 CRC patients  8 nurses | A Qualitative Multimethod Study | To explore patients and nurses' experiences of digital self-management support following participation in a remote patient monitoring intervention. | Semi-structured interview | Face-to-face interview | Approximately 40-60 minutes | Thematic analysis | 1. raising illness awareness through RPM technology, 2. establishing a mutual collaboration in self-management challenges 3. fostering a continued engagement in health behaviour change. |
