# Supplementary material for: Patients’ Experiences of Nurse-Led eHealth Interventions for Chronic Heart Failure: Qualitative Systematic Review and Meta-Synthesis
Source: J Med Internet Res. 2026 Jul 6;28:e82714. doi: 10.2196/82714 (PMC13335749; doi:10.2196/82714)
Supplement: Multimedia Appendix 3 [file jmir-v28-e82714-s003.docx]

# **Multimedia Appendix 3.** Results of the critical appraisal of the studies included

| Study | Q1 | Q2 | Q3 | Q4 | Q5 | Q6 | Q7 | Q8 | Q9 | Q10 | Level |
| --- | --- | --- | --- | --- | --- | --- | --- | --- | --- | --- | --- |
| Auton et al.[32] | U | Y | Y | Y | Y | U | N | Y | Y | Y | B |
| Birkhoff et al.[33] | Y | Y | Y | Y | Y | Y | N | Y | Y | Y | B |
| Buck et al.[34] | Y | Y | Y | Y | Y | Y | N | Y | Y | Y | B |
| Cajita et al.[35] | Y | Y | Y | Y | Y | Y | N | Y | Y | Y | B |
| Carter et al.[36] | Y | Y | Y | Y | Y | Y | N | Y | Y | Y | B |
| Fairbrother et al.[37] | Y | Y | Y | Y | Y | Y | N | Y | Y | Y | B |
| Gordon et al.[38] | Y | Y | Y | Y | Y | Y | N | Y | Y | Y | B |
| Jiang et al.[25] | Y | Y | Y | Y | Y | Y | N | Y | Y | Y | B |
| Jin et al.[39] | Y | Y | Y | Y | Y | Y | N | Y | Y | Y | B |
| Lan et al.[40] | U | Y | Y | Y | Y | U | Y | Y | Y | Y | B |
| Liu et al.[41] | U | Y | Y | Y | Y | Y | N | Y | Y | Y | B |
| Longhini et al.[42] | Y | Y | Y | Y | Y | Y | Y | Y | Y | Y | A |
| Lundgren et al.[43] | Y | Y | Y | Y | Y | Y | N | Y | Y | Y | B |
| Lyngå et al.[44] | U | Y | Y | Y | Y | Y | N | Y | Y | Y | B |
| Østrem et al. [45] | Y | Y | Y | Y | Y | Y | Y | Y | Y | Y | A |
| Säfström et al.[46] | U | Y | Y | Y | Y | U | N | Y | Y | Y | B |
| Sano et al.[47] | Y | Y | Y | Y | Y | Y | N | Y | Y | Y | B |
| Schmaderer et al.[26] | U | Y | Y | Y | Y | Y | N | Y | Y | Y | B |
| Son et al.[48] | U | Y | Y | Y | Y | Y | N | Y | Y | Y | B |
| Strandberg et al.[49] | Y | Y | Y | Y | Y | Y | N | Y | Y | Y | B |
| Vo et al.[27] | Y | Y | Y | Y | Y | Y | N | Y | Y | Y | B |
| Wali et al.[50] | Y | Y | Y | Y | Y | Y | N | Y | Y | Y | B |
| Study | Q1 | Q2 | Q3 | Q4 | Q5 | Q6 | Q7 | Q8 | Q9 | Q10 | Level |
| Wathne et al.[51] | Y | Y | Y | Y | Y | Y | N | Y | Y | Y | B |

Note:Y,yes; N,no; U, unclear; NP, not applicable

Q1.Is there congruity between the stated philosophical perspective and the research methodology?

Q2.Is there congruity between the research methodology and the research question or objectives?

Q3.Is there congruity between the research methodology and the methods used to collect data?

Q4.Is there congruity between the research methodology and the representation and analysis of data?

Q5.Is there congruity between the research methodology and the interpretation of results?

Q6.Is there a statement locating the researcher culturally or theoretically?

Q7.Is the influence of the researcher on the research,and vice-versa,addressed?

Q8.Are participants,and their voices,adequately represented?

Q9.Is the research ethical according to current criteria or,for recent studies,and is there evidence of ethical approval by an appropriate body?

Q10.Do the conclusions drawn in the research report flow from the analysis, or interpretation, of the data?
