# Supplementary material for: Patients’ Experiences of Nurse-Led eHealth Interventions for Chronic Heart Failure: Qualitative Systematic Review and Meta-Synthesis
Source: J Med Internet Res. 2026 Jul 6;28:e82714. doi: 10.2196/82714 (PMC13335749; doi:10.2196/82714)
Supplement: Multimedia Appendix 4 [file jmir-v28-e82714-s004.docx]

**Multimedia Appendix 4.** Mapping of Themes and Subthemes to Included Studies

| Themes | Sub-themes | Indicates that the theme/subtheme is present in the study |
| --- | --- | --- |
| 1.Patient Empowerment and Enhanced Self-Management | a.Improved Health Literacy and Disease Awareness | Auton et al.[32]  Jiang et al.[25]  Schmaderer et al.[26]  Son et al.[48]  Wathne et al.[51] |
|  | b.Strengthened Self-Efficacy and Confidence | Jiang et al.[25]  Østrem et al.[45]  Schmaderer et al.[26]  Son et al.[48] |
|  | c.Behavioral Change and Health Improvement | Fairbrother et al.[37]  Jiang et al.[25]  Longhini et al.[42]  Lundgren et al.[43]  Wathne et al.[51] |
| 2.Sense of Security and Continuity of Care Under Professional Support | a.Sense of Security from Remote Monitoring | Auton et al.[32]  Birkhoff et al.[33]  Lyngå et al.[44]  Sano et al.[47] |
|  | b.Continuity of Care and Collaborative Engagement | Auton et al.[32]  Carter et al.[36]  Fairbrother et al.[37]  Gordon et al.[38]  Jiang et al.[25]  Wathne et al.[51] |
|  | c.Communication and Relationship Building | Auton et al.[32]  Birkhoff et al.[33]  Jiang et al.[25]  Østrem et al.[45]  Sano et al.[47]  Wali et al.[50] |
| 3.Variations in Acceptance and Emotional Responses | a.Positive Acceptance and Motivation | Auton et al.[32]  Cajita et al.[35]  Carter et al.[36]  Vo et al.[27] |
|  | b.Negative Attitudes and Psychological Resistance | Cajita et al.[35]  Jin et al.[39]  Lan et al.[40]  Liu et al.[41] |
|  | c.Individual Differences and Applicability | Cajita et al.[35]  Lan et al.[40]  Liu et al.[41] |
| Themes | Sub-themes | Indicates that the theme/subtheme is present in the study |
| 3.Variations in Acceptance and Emotional Responses | c.Individual Differences and Applicability | Strandberg et al.[49] |
| 4.Barriers and Challenges in Implementing eHealth Interventions | a.Technological Usability and User Experience | Buck et al.[34]  Cajita et al.[35]  Carter et al.[36]  Fairbrother et al.[37]  Gordon et al.[38]  Jiang et al.[25]  Lundgren et al.[43]  Säfström et al.[46] |
|  | b.Technical Issues and Usage Barriers | Buck et al.[34]  Jin et al.[39]  Lundgren et al.[43]  Wali et al.[50] |
|  | c.Concerns About Sustainability | Cajita et al.[35]  Jin et al.[39]  Wali et al.[50] |
