# Supplementary material for: Patients’ Experiences of Nurse-Led eHealth Interventions for Chronic Heart Failure: Qualitative Systematic Review and Meta-Synthesis
Source: J Med Internet Res. 2026 Jul 6;28:e82714. doi: 10.2196/82714 (PMC13335749; doi:10.2196/82714)
Supplement: Multimedia Appendix 5 [file jmir-v28-e82714-s005.docx]

**Multimedia Appendix 5.** Full ConQual Assessment of the Synthesized Findings

| Synthesized findings | Contributing studies | Dependability | Credibility | ConQual rating |
| --- | --- | --- | --- | --- |
| Theme 1. Patient Empowerment and Enhanced Self-Management | 9 studies [25,26,32,37,42,43,45,48,51] | High | Moderate | Moderate |
| 1a. Improved Health Literacy and Disease Awareness | 5 studies [25,26,32,48,51] | High | High | High |
| 1b. Strengthened Self-Efficacy and Confidence | 4 studies [25,26,45,48] | High | Moderate | Moderate |
| 1c. Behavioral Change and Health Improvement | 5 studies [25,37,42,43,51] | High | Moderate | Moderate |
| Theme 2. Sense of Security and Continuity of Care Under Professional Support | 11 studies [25,32,33,36-38,44,45,47,50,51] | High | Moderate | Moderate |
| 2a. Sense of Security from Remote Monitoring | 4 studies [32,33,44,47] | High | High | High |
| 2b. Continuity of Care and Collaborative Engagement | 6 studies [25,32,36-38,51] | High | Moderate | Moderate |
| 2c. Communication and Relationship Building | 6 studies [25,32,33,45,47,50] | High | Moderate | Moderate |
| Theme 3. Variations in Acceptance and Emotional Responses | 8 studies [27,32,35,36,39-41,49] | High | Moderate | Moderate |
| 3a. Positive Acceptance and Motivation | 4 studies [27,32,35,36] | High | Moderate | Moderate |
| 3b. Negative Attitudes and Psychological Resistance | 4 studies [35,39-41] | High | Moderate | Moderate |
| 3c. Individual Differences and Applicability | 4 studies [35,40,41,49] | High | Moderate | Moderate |
| Theme 4. Barriers and Challenges in Implementing eHealth Interventions | 10 studies [25,34-39,43,46,50] | High | Moderate | Moderate |
| 4a. Technological Usability and User Experience | 8 studies [25,34-38,43,46] | High | Moderate | Moderate |
| 4b. Technical Issues and Usage Barriers | 4 studies [34,39,43,50] | High | High | High |
| 4c. Concerns About Sustainability | 3 studies [35,39,50] | High | Moderate | Moderate |

**Note:** Dependability was assessed according to the methodological quality of the contributing studies, particularly the congruity between the stated philosophical perspective, methodology, research questions, data collection, data analysis, and interpretation. Credibility was assessed according to the degree of support between the original participant quotations and the synthesized findings. Although the contributing studies generally demonstrated acceptable methodological quality, several studies provided limited reporting on researcher reflexivity and the influence of the researcher on the research process. Three subthemes, namely improved health literacy and disease awareness, sense of security from remote monitoring, and technical issues and usage barriers, were supported by direct and unequivocal participant quotations and were therefore rated as high confidence. Other synthesized themes and subthemes were supported by a combination of unequivocal and credible evidence rather than exclusively unequivocal evidence. Based on these considerations, the final ConQual ratings for the synthesized themes and subthemes ranged from moderate to high.
